# The Importance of Ditches and Canals in Global Inland Water CO_2_ and N_2_O Budgets

**DOI:** 10.1111/gcb.70079

**Published:** 2025-03-07

**Authors:** Teresa Silverthorn, Joachim Audet, Chris D. Evans, Judith van der Knaap, Sarian Kosten, José Paranaíba, Quinten Struik, Jackie Webb, Wenxin Wu, Zhifeng Yan, Mike Peacock

**Affiliations:** ^1^ Department of Geography and Planning, School of Environmental Sciences University of Liverpool Liverpool UK; ^2^ Department of Ecoscience Aarhus University Aarhus Denmark; ^3^ UK Centre for Ecology & Hydrology Bangor UK; ^4^ Department of Ecology, Radboud Institute for Biological and Environmental Sciences Radboud University Nijmegen the Netherlands; ^5^ School of Agriculture and Environmental Science University of Southern Queensland Toowoomba Queensland Australia; ^6^ Centre for Sustainable Agricultural Systems University of Southern Queensland Toowoomba Queensland Australia; ^7^ Ecohydrology Research Group, Department of Earth and Environmental Sciences University of Waterloo Ontario Canada; ^8^ Institute of Surface Earth System Science, School of Earth System Science Tianjin University Tianjin China; ^9^ Department of Aquatic Sciences and Assessment Swedish University of Agricultural Sciences Uppsala Sweden

**Keywords:** biogeochemistry, carbon dioxide, freshwater ecosystems, greenhouse gas emissions, nitrous oxide

## Abstract

Ditches and canals are omitted from global budgets of inland water emissions, despite research showing them to be emitters of greenhouse gases (GHGs). Here, we synthesize data across climate zones and land use types to show, for the first time, that global ditches emit notable amounts of carbon dioxide (CO_2_) and nitrous oxide (N_2_O). Ditches had higher per‐area emissions of CO_2_ and N_2_O than ponds, lakes, and reservoirs, likely due to high nutrient inputs. Preliminary upscaling showed that the inclusion of ditches would increase global inland water CO_2_ emissions by 0.6%–1% and N_2_O emissions by 3%–9%. Trophic state and climate influenced N_2_O emissions, while CO_2_ emissions had complex drivers difficult to disentangle at the global scale. This research highlights the importance of including ditches in global inland water GHG budgets and informs more accurate reporting of anthropogenic emissions in national inventories.

## Graphical abstract

We synthesized data across global climate zones to show, for the first time, that global ditches and canals emit notable amounts of carbon dioxide and nitrous oxide, at higher per area rates than ponds, lakes, and reservoirs. Our upscaling shows that the inclusion of ditches would increase global inland water greenhouse gas budgets, demonstrating the importance of accounting for ditches in efforts to measure and reduce anthropogenic emissions.

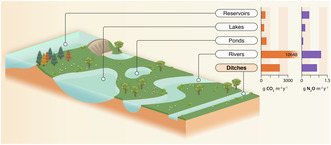

## 1 Introduction

Inland waters (e.g., rivers, streams, lakes, ponds, and reservoirs) are active sites of biogeochemical cycling and outgas (as carbon dioxide [CO_2_] or methane [CH_4_]) 3.9 Pg of carbon, or ~75% of the carbon they receive from terrestrial ecosystems (Drake et al. 2018). In parallel, inland waters contribute ~7% to total global nitrous oxide (N_2_O) emissions (Wang et al. 2023). The cycling of carbon and nitrogen in inland waters is altered by climate change and agricultural and urban expansion, often resulting in an increase in greenhouse gas (GHG) emissions to the atmosphere (Battin et al. 2023). Anthropogenic nitrogen inputs (notably from agricultural fertilizer applications) (Yao et al. 2020) and global warming (Velthuis and Veraart 2022) have resulted in an increase in aquatic N_2_O emissions. Aquatic CO_2_ export is increasing in high latitudes and the tropics, likely due to augmented dissolved organic matter concentrations (i.e., freshwater “browning”) (Lapierre et al. 2013) and atmospheric CO_2_ fertilization (Hastie et al. 2021), respectively.

In order to mitigate GHG emissions from freshwaters, a full and accurate accounting of sources, and an understanding of how global change effects them, is required. However, noticeably absent from inland water GHG budgets are drainage ditches and canals. Ditches and canals (hereafter: ditches) are human‐made waterways constructed to serve a variety of purposes (Clifford and Heffernan 2018), such as improving the productivity of wet soils (Simola et al. 2012), reclaiming flooded land (Haasnoot et al. 2020), transporting water for irrigation (Palmia et al. 2021), and redistributing urban runoff (McPhillips et al. 2016). Although ditches are typically constructed to move water, some dry out temporarily and are referred to here as non‐perennial ditches. Because of the wide variation in ditch function, morphology, and water status it is difficult to strictly define “ditches,” but a rough catch‐all is that ditches are human‐constructed linear depressions, that generally carry water for at least some of the time. Due to their utility and diversity of function, the cumulative global extent of ditches is large, but poorly quantified (Peacock, Audet, Bastviken, Futter, et al. 2021). Estimates of cumulative ditch length at regional scales can be especially large (rivalling stream and river length) for heavily drained countries such as the Netherlands (300,000 km) (Koschorreck et al. 2020), the United Kingdom (600,000 km) (Brown et al. 2006), as well as parts of Southeast Asia (Dadap et al. 2021), China (Wu et al. 2023), and the United States (Clifford and Heffernan 2023).

Ditches can emit large amounts of GHGs, often more per unit area than other inland waters (Outram and Hiscock 2012; Peacock, Audet, Bastviken, Cook, et al. 2021), and frequently have higher CH_4_ emissions than surrounding terrestrial environments (Evans et al. 2016). Commonly found in intensively managed agricultural and urban landscapes, ditches often receive high nutrient inputs (Stets et al. 2020). This, coupled with low flow velocities, can result in conditions ideal for CH_4_ production through methanogenesis (Wu et al. 2023) and N_2_O production through denitrification (Webb et al. 2021). Because ditches are often constructed to drain the surrounding terrestrial landscape they can have high land‐water connectivity (Levavasseur et al. 2015). As a result, they can receive substantial lateral inputs of organic carbon, nutrients and dissolved GHGs (Rocher‐Ros et al. 2023), ultimately contributing to GHG supersaturation and emission (Hotchkiss et al. 2015).

Shallow waters and slow flows in ditches create favorable conditions for vegetation development (Needelman et al. 2007) and intermittent drying (Gallo et al. 2014), both of which can significantly affect the GHG balance. The presence of vegetation can reduce N_2_O and CO_2_ emissions through plant nitrogen (Zhang et al. 2016) and photosynthetic uptake (Jeppesen et al. 2016), respectively. Unnatural hydrological regimes due to fluctuating human demands for water (e.g., for irrigation) (Macdonald et al. 2016), result in a high probability of ditches intermittently drying (Herzon and Helenius 2008). Drying can cause notable CO_2_ emissions due to oxygenation of sediments (Keller et al. 2020) but reduce CO_2_ emissions following flow resumption (Silverthorn et al. 2024). Drying can also trigger pulses of N_2_O upon ditch sediment rewetting (Gallo et al. 2014).

Despite research pointing to ditches as large emitters of GHGs, their total (CH_4_, CO_2_, and N_2_O) emissions are still not explicitly accounted for in national GHG inventories. Unlike emissions from other inland waters (e.g., lakes and rivers), which are considered a natural component of the global GHG budget, ditches are anthropogenic features, and therefore their emissions should be accounted for in regional, national, and international inventory reports (Lovelock et al. 2019). We recommend that ditch emissions are considered as anthropogenic “flooded land” emissions (similar to emissions from reservoirs and constructed ponds), in line with the approach taken in the 2019 Intergovernmental Panel on Climate Change (IPCC) Wetlands chapter (Lovelock et al. 2019). Guidelines for accounting for ditch CH_4_ emissions were included for organic soils in the 2013 IPCC Wetlands Supplement (IPCC 2014), and for all soils in the 2019 IPCC Refinement (Lovelock et al. 2019). These guidelines take the form of emission factors (in kg CH_4_ ha^−1^ yr.^−1^) which can be multiplied by the cumulative ditch surface area (within a landscape, region, or country) to calculate total ditch emissions. However, there are still no similar guidelines for CO_2_ and N_2_O. Although, in the 2019 Refinement, ditch N_2_O emissions are considered as indirect emissions from nitrate leaching from land to water based on conversion factors according to rates of fertilizer and manure inputs.

Research has focused on CH_4_ due to favorable conditions for methanogenesis in ditches, with global CH_4_ emissions from ditches estimated at 0.2% to 3% of total global anthropogenic CH_4_ emissions (Peacock, Audet, Bastviken, Futter, et al. 2021). However, CO_2_ and N_2_O emissions can also be significant. For instance, CO_2_ flux accounted for > 98% of total GHG flux from drainage ditches in an abandoned peat extraction area in Estonia (Järveoja et al. 2016), and 89% from tropical peatland ditches draining oil palm plantations (Manning et al. 2019). In an agricultural catchment in England, N_2_O emissions from ditches accounted for 31% of the total N_2_O budget, despite covering less than 4% of the total catchment area (Outram and Hiscock 2012). While research on CO_2_ and N_2_O emissions from ditches is increasing, a comprehensive global‐scale synthesis is lacking.

Here, we aim to highlight the potential importance of the inclusion of ditches in global freshwater GHG budgets and to provide data to support the potential future refinement of ditch CO_2_ and N_2_O emission reporting guidelines. Our objective was to address these knowledge gaps by collating the available data on the magnitudes and drivers of CO_2_ and N_2_O fluxes in ditches globally. We expected higher GHG emissions per unit surface area from ditches when compared to other inland waters due to high nutrient inputs and close land‐water connectivity. We expected the highest CO_2_ and N_2_O emission rates in intensively managed systems with high nutrient (e.g., agricultural/urban land uses) and carbon inputs (e.g., catchments draining organic soils). The presence of in‐stream vegetation was expected to result in lower CO_2_ emissions due to uptake via primary production and lower N_2_O emissions due to the associated nutrient demand. We also expected that non‐perennial ditches (i.e., those which are periodically dry) would have high dry‐condition emissions but lower aquatic emissions than perennially water‐filled ditches due to the negative impacts of desiccation on aquatic microbial communities.

## 2 Materials and Methods

### 2.1 Data Compilation

We conducted a bibliographic search through the Web of Science database using search terms targeting studies measuring ditch CO_2_ and N_2_O emissions, published until January 2024 (see Table S1 for a full description of the search terms). The resulting 954 results were checked for relevance, with 38 included in our final dataset. We accepted studies that measured in situ CO_2_ and/or N_2_O emissions from ditches and canals using either floating chamber flux measurements or flux estimates derived from dissolved GHG concentrations. To identify additional studies, we uploaded our initial bibliography into Litmap, an online research platform that identifies relevant research studies through citations and references. We also screened the reference lists of the identified articles to find further relevant studies. In addition, the study coauthors and other researchers provided their personal reference lists of relevant studies, as well as three unpublished datasets. Three non‐English language articles (Russian and Chinese) were translated by native speakers, who aided in the data extraction. We also included studies from the coauthors' personal reference lists where sites were referred to as streams or rivers in the text, but contact with the authors, site descriptions, and/or maps confirmed that these were in fact ditches. However, it is likely that there are studies not captured in our review that referred to ditches as streams and were therefore not captured in our literature search. In total, we had 77 unique studies (including journal articles, theses, reports, and unpublished datasets) in our final dataset.

We collected a suite of information about the location, climate, ditch physical characteristics, GHG emissions (CO_2_ and/or N_2_O, as well as CH_4_ if it was also reported), and water quality variables for each study, when available. See Table S2 for a full list of the information collected. When values were not reported in‐text, we used WebPlotDigitizer v4 (https://automeris.io/) to extract data from figures. We disaggregated data from a single study if the sampled ditches had different land use, trophic status, catchment soil type, hydrological regime, and/or vegetation presence vs. absence. As a result of this data partitioning, our final dataset contains a total of 119 unique ditch sites with 99 observations of CO_2_ and 56 observations of N_2_O (Silverthorn 2025). The database also has 94 observations of diffusive CH_4_ and 20 observations of ebullitive CH_4_. We categorized the data into four land use categories based on the Corine Land Cover classification system: agriculture, natural/forest, urban, and wetland (European Environment Agency 2012). Natural/forest land use indicates ditches in areas that may be relatively undisturbed or near‐natural (e.g., a forest or peatland with a ditch in it, but where no further ecosystem modification/degradation has taken place), or may be managed land (e.g., forest plantations, forests drained to increase productivity). We categorized the data into four main climate categories based on the Köppen Geiger climate classification: continental, temperate, arid, and tropical (Köppen et al. 2011). We also categorized the data into four categories of trophic state: hypereutrophic, eutrophic, mesotrophic, and oligotrophic using available nutrient concentration data (Smith et al. 1999). We further classified the data by catchment soil type: mineral or organic; and hydrological regime: perennial or non‐perennial (dry or water‐filled). See Text S1 for more details on how we classified the data into each category and interpolated missing values.

We calculated annual GHG fluxes following Peacock, Audet, Bastviken, Futter, et al. (2021). Briefly, if authors did not report annual GHG fluxes, we multiplied the daily flux (converted to grams of CO_2_, N_2_O, or CH_4_, if necessary) by the growing season length for sites with seasonal ice/snow cover, otherwise we multiplied the daily flux by 365. The temporal variability of GHG sampling varied, with frequencies ranging from continuous monitoring sensors, weekly, monthly, once per season, or once per year. We recognize that our calculations may over‐ or underestimate fluxes if the GHG sampling did not cover a representative range of seasonality. Moreover, the majority of studies measure daytime emissions, thus we refine our CO_2_ estimate by accounting for night‐time emissions (Gómez‐Gener et al. 2021). In addition to ditch surface area and temporal sampling variability, there is uncertainty associated with our upscaling due to the geographic data gaps in Africa and South America.

### 2.2 Data Analysis

All statistical analyses were conducted using the statistical software R, version 4.4.0 (R Core Team 2024), using base R functions unless otherwise specified. Significance was accepted at *p* < 0.05. We tested for differences in CO_2_ and N_2_O fluxes between the categorical variables (i.e., GHG sampling method, land use type, climate zone, trophic state, soil type, hydrological regime, and vegetation presence) using the nonparametric Kruskal–Wallis rank sum test (*kruskal.test* function) or Mann–Whitney *U* test (*wilcox. test* function), due to the non‐normal distribution of the data (Hollander et al. 2013). We used the pairwise Wilcoxon rank sum test (*pairwise.wilcox.test* function) to calculate post hoc comparisons between group levels, with corrections for multiple testing. We found no significant differences in CO_2_ and N_2_O emissions between the GHG sampling methods (floating chamber vs. estimation from dissolved concentrations; Figure S1) from independent studies using either method, supporting the inclusion of data based on both methods in this synthesis.

We plotted the relationships between CO_2_ and N_2_O and the quantitative explanatory variables (latitude, meters above sea level [masl], mean annual temperature [MAT], mean annual precipitation [MAP], ditch width, water depth, velocity, discharge, dissolved oxygen [DO], pH, electrical conductivity [EC], dissolved organic carbon [DOC], total phosphorus [TP], total nitrogen [TN], chlorophyll‐*a* [chl‐*a*], nitrate nitrogen [NO_3_‐N]), and carbon:nitrogen for a visual check of any potential nonlinear relationships. Subsequently, we tested for significant monotonic relationships between CO_2_ and N_2_O and these variables using Spearman rank correlation coefficients (Zuur et al. 2007).

To calculate the relative contribution of each GHG in terms of CO_2_ equivalents, we used a subset of studies (*n* = 22) that had measurements of all three GHGs. For CH_4_ we considered the total (diffusive + ebullitive emissions), although only two of the 22 studies measured ebullitive emissions. We used a Global Warming Potential over a 100‐year horizon (GWP_100_) of 27 for CH_4_ and 273 for N_2_O (Forster et al. 2021). We used a Sustained Global Warming Potential (SGWP_100_) for emissions (45 for CH_4_, 270 for N_2_O) and Sustained Global Cooling Potential (SGCP_100_) for uptake (203 for CH_4_, 349 for N_2_O), over the same time horizon (Neubauer and Megonigal 2015).

To upscale to global ditch CO_2_ and N_2_O emissions, we used the estimated surface area of ditches (5,353,000 ha) from Peacock, Audet, Bastviken, Futter, et al. (2021). Briefly, this ditch surface area estimate was calculated using the total global drained land area (for forestry and agriculture) and the average proportion of this drained area covered by ditches and canals (so‐called Frac_ditch_), taken from literature values. In Peacock, Audet, Bastviken, Futter et al. (2021), a set Frac_ditch_ of 3% was used, with lower and upper values of 1% and 5% used to give a surface area uncertainty range of 1,420,000—10,700,000 ha. This is a conservative estimate as it does not include most irrigation, estuarine, or urban canals given that it only considers ditches on drained lands. For the global emission estimates we used the mean values from our literature synthesis with a 95% confidence interval (CI) calculated using the *t.test* function. We compared our estimate to estimates of total global anthropogenic N_2_O emissions of 6.5 Tg N yr.^−1^, global inland water N_2_O emissions of 0.5 Tg N yr.^−1^ (Tian et al. 2024), total global anthropogenic CO_2_ emissions of 11.1 Pg C yr.^−1^ (Friedlingstein et al. 2023), and global inland water CO_2_ emissions of 3.9 Pg C yr.^−1^ (Drake et al. 2018).

## 3 Results

### 3.1 Magnitude and Drivers of Global Ditch CO_2_ and N_2_O Emissions

**FIGURE 1:**
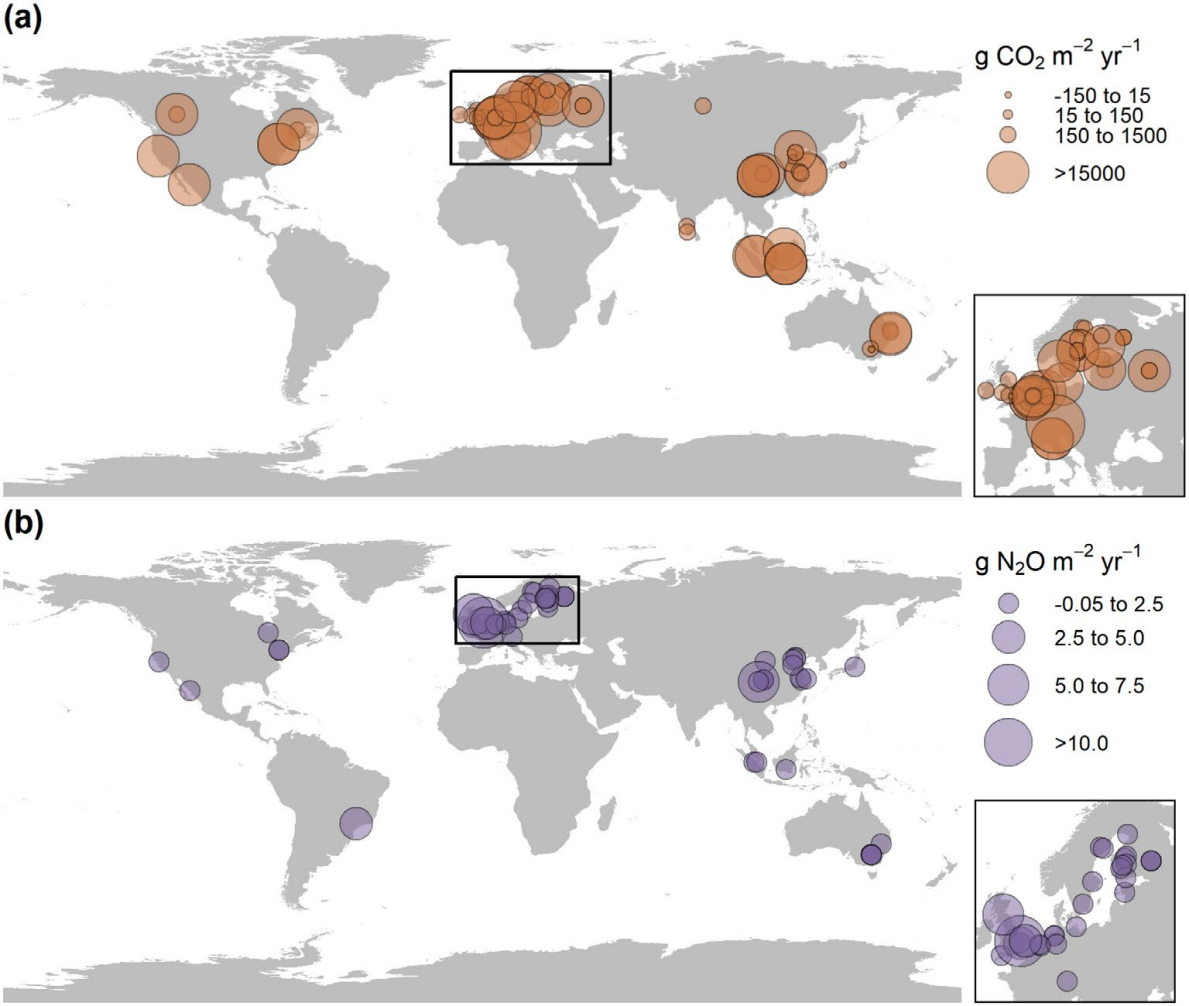
Global distribution of carbon dioxide (a) and nitrous oxide (b) emissions from ditches, where point size indicates flux magnitude. The inset maps magnify the high sampling intensity in Europe.

**FIGURE 2:**
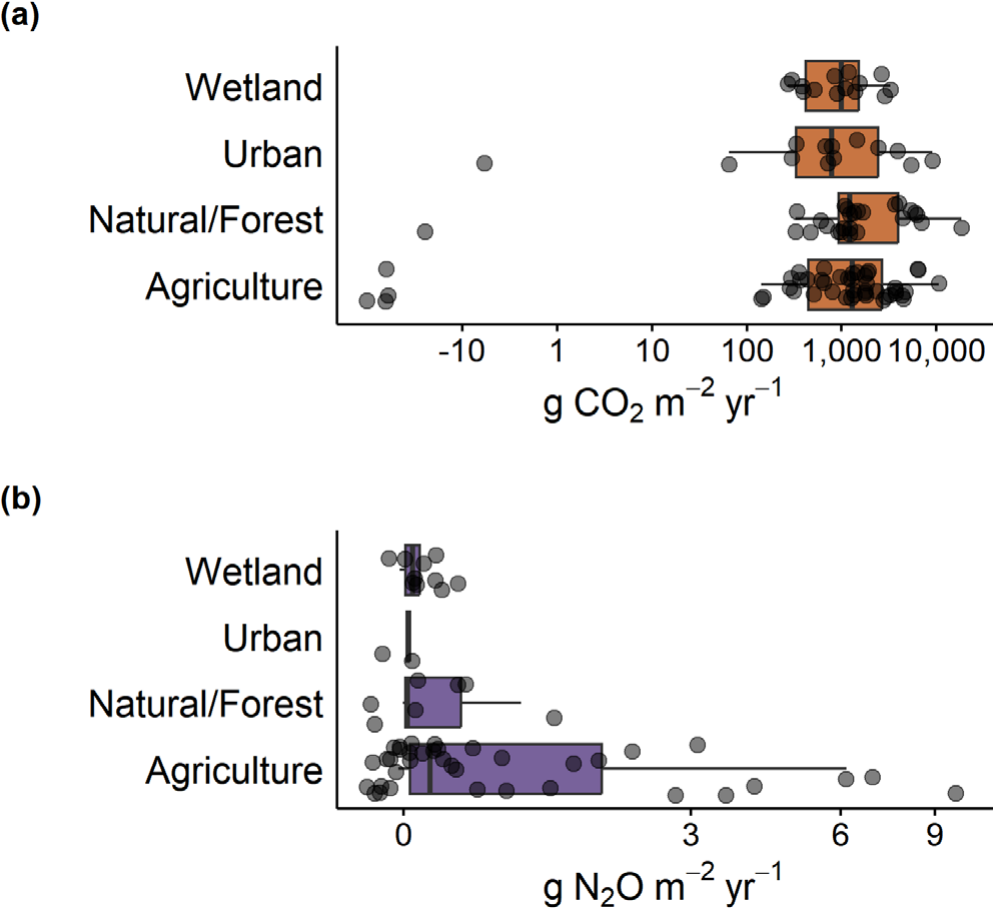
Carbon dioxide (a) and nitrous oxide (b) emissions from global ditches did not significantly differ between land use (CO_2_: *p* = 0.54 and N_2_O: *p* = 0.11, Kruskal–Wallis test).

**FIGURE 3:**
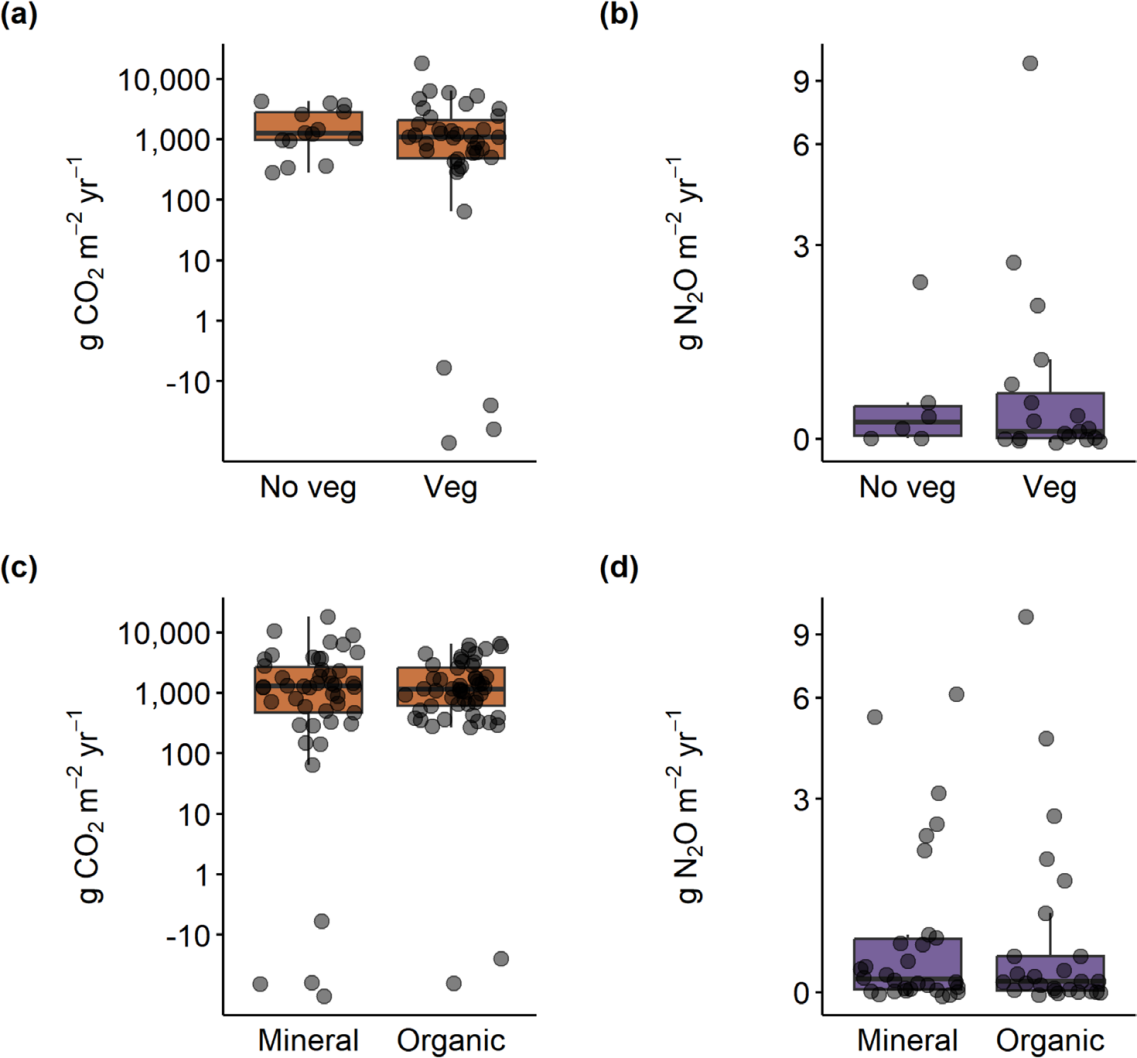
Carbon dioxide and nitrous oxide emissions from global ditches did not differ by the presence (“Veg”) or absence (“No veg”) of ditch vegetation (a, b; CO_2_: *p* = 0.42 and N_2_O: *p* = 0.66, Mann–Whitney *U* test) nor by soil type (c, d; CO_2_: *p* = 0.89 and N_2_O: *p* = 0.67, Mann–Whitney *U* test).

**FIGURE 4:**
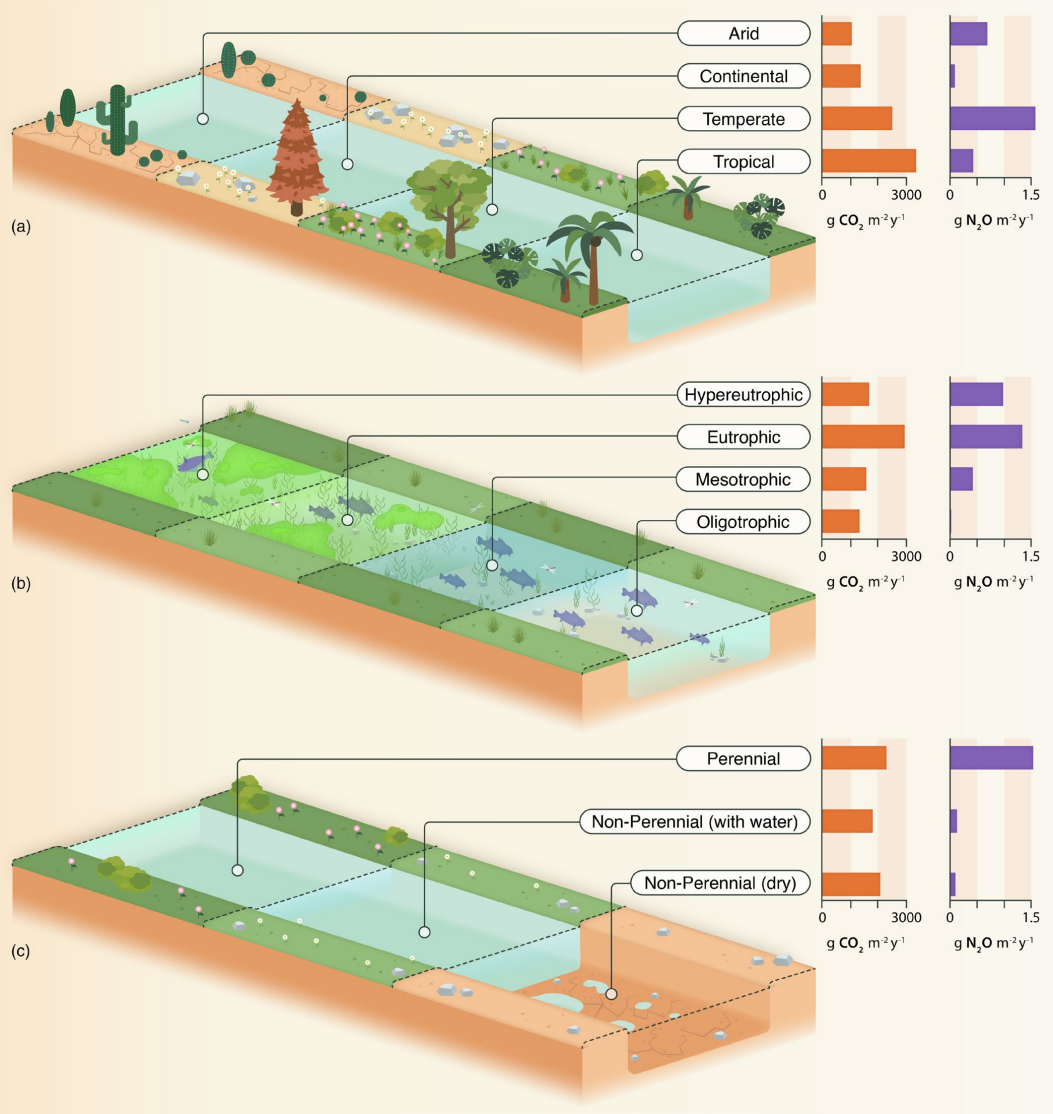
Carbon dioxide emissions (g CO_2_ m^−2^ yr.^−1^) in orange and nitrous oxide emissions (g N_2_O m^−2^ yr.^−1^) in purple from global ditches by climate (a), trophic state (b), and hydrological regime (c). See Table S4 for source data. Figure designed by Miranta Kouvari from Science Graphic Design.

The majority of ditches were a net source of CO_2_ (mean ± SD: 2060 ± 2620 g CO_2_ m^−2^ yr.^−1^) and N_2_O (0.892 ± 1.83 g N_2_O m^−2^ yr.^−1^) to the atmosphere (Figure 1). Ditches were net annual sinks of CO_2_ (−63.4 ± 27.4 g CO_2_ m^−2^ yr.^−1^) and N_2_O (−0.0217 ± 0.0163 g N_2_O m^−2^ yr.^−1^) in only five and six cases, respectively. Emissions of CO_2_ did not differ consistently between land use type, climate zone, trophic state, hydrological regime, and vegetation presence (Figures 2, 3, 4). Emissions of N_2_O did not differ between land use type (Figure 2b) and vegetation presence (Figure 3a). Although DOC concentrations were significantly higher in ditches draining organic than mineral catchments (*p* < 0.0001, Mann–Whitney *U* test; Figure S2), there were no differences in CO_2_ or N_2_O emissions between the two catchment soil types (Figure 3c,d). Ditches in temperate climates had higher N_2_O emissions than those in continental climates (*p* < 0.0001, Wilcoxon test; Figure 4a). N_2_O emissions were consistently low in continental climates, where MAT is low (typically < 10°C; Figure S3). Hypereutrophic and eutrophic ditches both had higher N_2_O emissions than mesotrophic and oligotrophic ditches (*p* = 0.001, Kruskal–Wallis test; Figure 4b). Perennially water‐filled ditches had higher N_2_O emissions than non‐perennial ditches (*p* = 0.001, Mann–Whitney U test; Figure 4c). Dry ditch N_2_O emissions were on average 16 times lower than emissions from perennial ditches, although the small sample size of dry ditches (*n* = 3) precluded this difference from being significant (Figure 4c).

**FIGURE 5:**
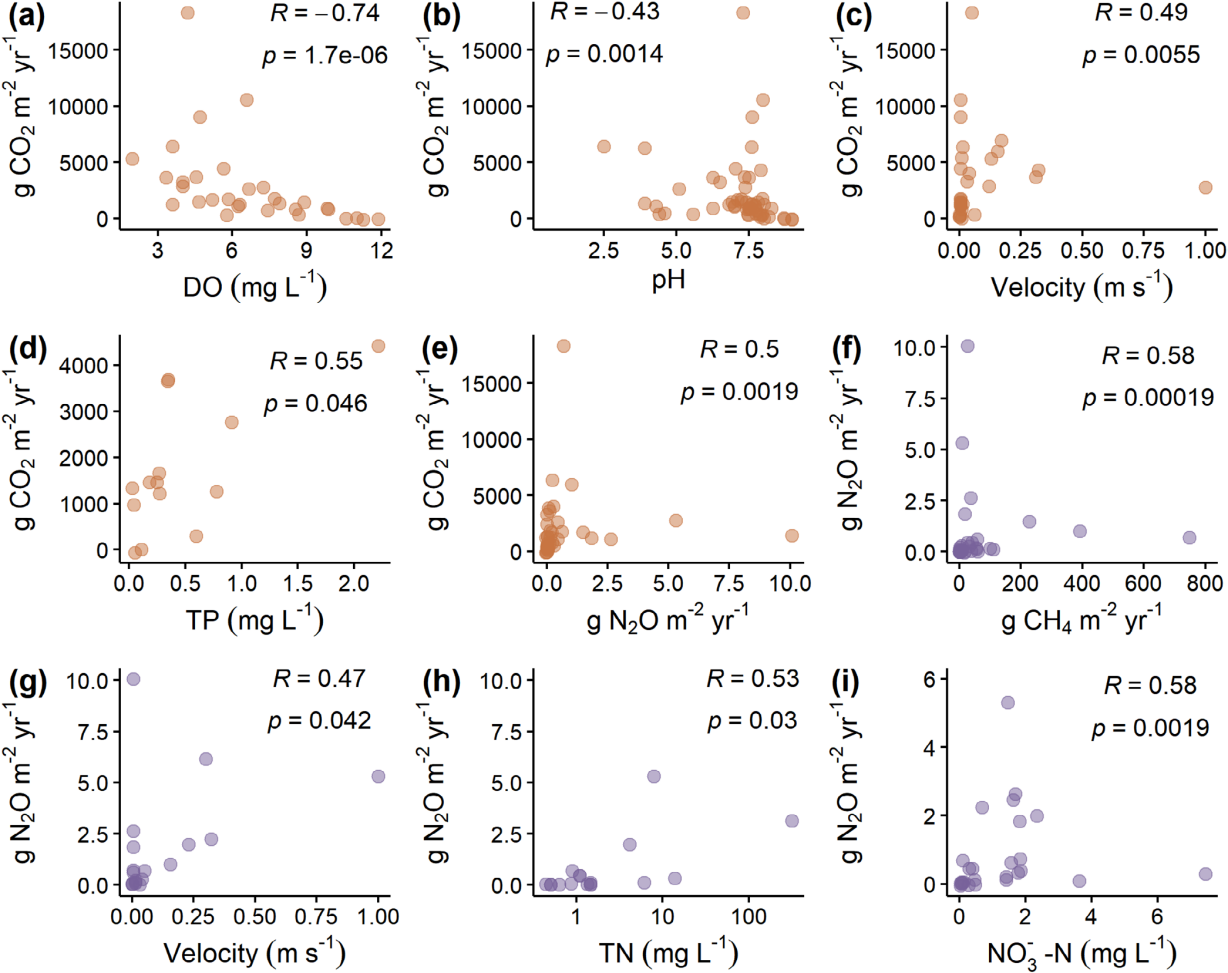
Environmental variables with significant Spearman correlations (*R*) illustrating strong (*R* > 0.6) or moderate (*R* > 0.4) correlations with carbon dioxide (a–e) and nitrous oxide (f–i) emissions.

Among the quantitative environmental variables, CO_2_ emissions were strongly correlated with DO concentrations (Spearman *R [R]*: −0.74, *p* < 0.0001), and moderately correlated with pH (*R* = −0.43, *p* = 0.001), water velocity (*R* = 0.49, *p* = 0.006), N_2_O emissions (*R* = 0.50, *p* = 0.002), and TP concentrations (*R* = 0.55, *p* = 0.046) (Figure 5a–e). For N_2_O, emissions were moderately correlated with diffusive CH_4_ emissions (*R* = 0.58, *p* = 0.0001), water velocity (*R* = 0.47, *p* = 0.04), TN concentrations (*R* = 0.53, *p* = 0.03), and NO_3_^−^‐N concentrations (*R* = 0.58, *p* = 0.002) (Figure 5f–i).

### 3.2 Relative Warming Impacts of CO_2_ , CH_4_ and N_2_O Emissions From Ditches

In terms of emissions in CO_2_ equivalents, based on GWP_100_, CO_2_ had the largest contribution (51%), followed by CH_4_ (43.5%), and N_2_O (5.5%) (Table S3). Based on SGWP_100_/SGCP_100_, CH_4_ had the largest contribution (54%), followed by CO_2_ (41%), and N_2_O (5%) (Table S3).

### 3.3 Global Ditch CO_2_ and N_2_O Emissions

**FIGURE 6:**
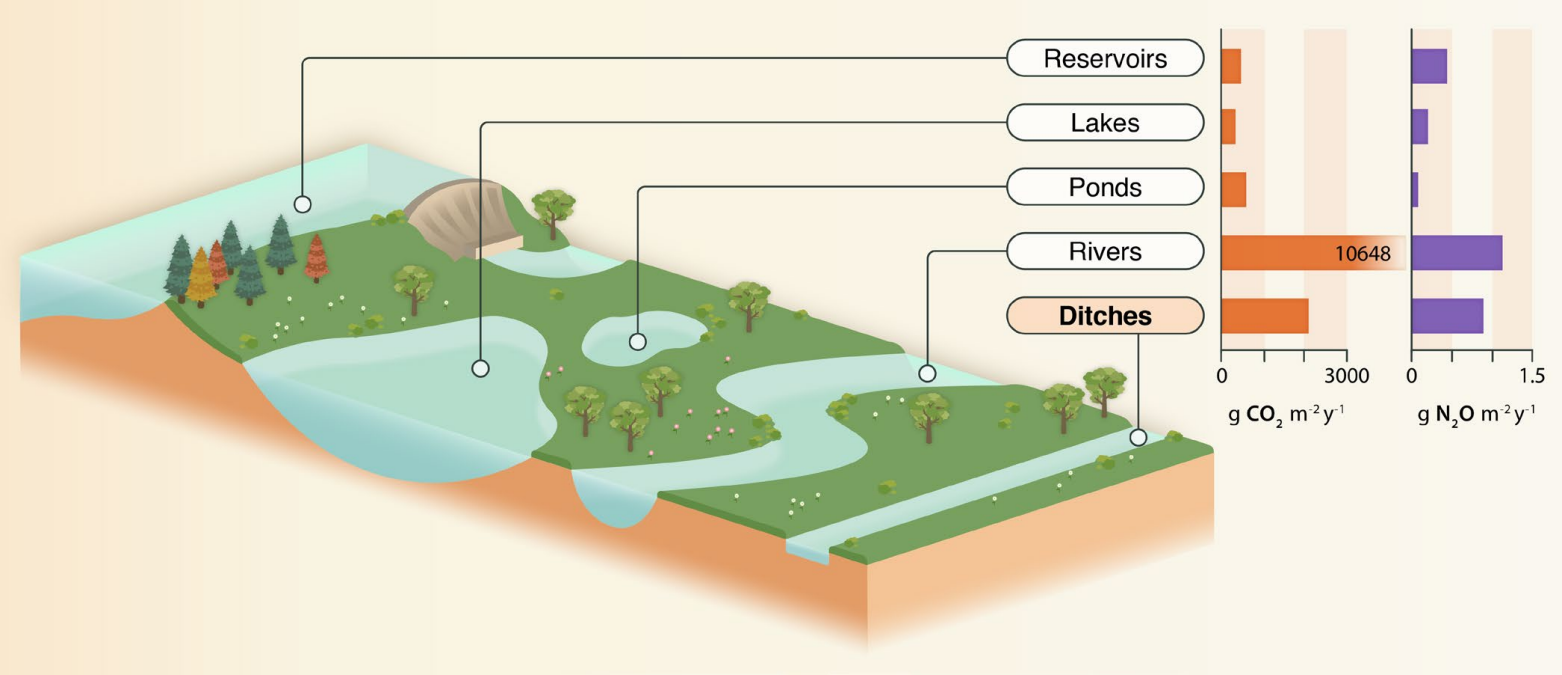
Carbon dioxide emissions (g CO_2_ m^−2^ yr.^−1^) in orange and nitrous oxide emissions (g N_2_O m^−2^ yr.^−1^) in purple from ditches compared to other inland waters. See Table S5 for source data. Figure designed by Miranta Kouvari from Science Graphic Design.

Global ditches emit an estimated 30.0 Tg C yr.^−1^ (95% CI: 22.4–37.7 Tg C yr.^−1^) as CO_2_ and 0.03 Tg N yr.^−1^ as N_2_O (95% CI: 0.01–0.05 Tg N yr.^−1^). These values represent 0.2 to 0.3% (mean: 0.3%) and 0.2 to 0.7% (mean: 0.5%) of global anthropogenic CO_2_ and N_2_O emissions, respectively. The addition of ditch emissions would increase global inland water CO_2_ emission estimates by 0.6% to 1% (mean: 0.8%) and N_2_O emissions by 3% to 9% (mean: 6%). Ditches had, on average, lower CO_2_ and N_2_O emissions than reported values for rivers and streams, but higher emissions than ponds, lakes, and reservoirs (Figure 6 and Table S5).

## 4 Discussion

Our analysis, based on 119 unique ditch sites, encompassing all major climate zones (besides polar) and land use types, shows that ditches are persistent emitters of CO_2_ and N_2_O, but with variable relationships to environmental drivers. Ditches have higher CO_2_ and N_2_O emissions per unit area compared to ponds, lakes, and reservoirs; this is likely due to their shorter water residence times resulting in lower primary productivity (Sarkar and Kumar 2024) and incomplete denitrification (Maavara et al. 2019), as well as higher average nutrient inputs given their predominantly agricultural and urban landscape contexts (Stets et al. 2020). Lower areal ditch CO_2_ and N_2_O emissions compared to rivers and streams, is likely due to the higher gas transfer velocity (driven by high slope and stream flow velocity) in the latter, which promotes rapid degassing (Raymond et al. 2013). Given the uncertainty associated with the global extent of ditches, as well as limited flux data, our calculations that the inclusion of ditches would increase global inland water CO_2_ budgets by 0.6% to 1% and global N_2_O budgets by 3% to 9% serve as initial first‐order estimates. These values suggest that ditches are important GHG emitters, that should be included in future global inland water GHG budgets.

### 4.1 Accounting for Global Ditch CO_2_ and N_2_O Emissions

Care should be taken to avoid double‐counting ditch emissions as inland water, agriculture, and/or drained wetland emissions. For example, ditch N_2_O emissions are accounted for in the IPCC method for quantifying indirect N_2_O emissions: incidental emissions produced from inland waters as a result of nitrogen runoff and leaching from agricultural land. Thus, there is some chance of double counting if ditch emissions are inventoried this way. However, the inclusion of ditches in the IPCC emission factor guidelines does not yet translate to the inclusion of ditch N_2_O emissions in the global N_2_O budget, which focuses on rivers, reservoirs, and estuaries (Maavara et al. 2019; Tian et al. 2024). Furthermore, ditch N_2_O emissions are poorly represented by the indirect emission factor model, which groups ditches into “groundwater and surface drainage,” wherein ditches were found to have a significantly different emission factor compared with groundwater and subsurface drains, and therefore deserving of their own emission factor in the IPCC methodology (Webb et al. 2021). Because no accounting of ditch CO_2_ emissions currently takes place under IPCC guidelines (either under the 2019 Refinement or 2013 Wetlands Supplement) we assume there is no scope for double‐counting. Similarly to N_2_O, ditch CO_2_ emissions are not currently considered in the global CO_2_ budget (Drake et al. 2018; Friedlingstein et al. 2023). The areal extent of fluvial systems used in most global inland water GHG upscaling efforts uses modelling based on topography (Lehner et al. 2008). Given that ditches are artificially created, they may not follow natural flow lines, and therefore many will not be captured in these models. Moreover, the most commonly used fluvial database has a lower bound of a drainage area of 10 km^2^ or a mean annual discharge of 0.1 m^3^ s^−1^ (Grill et al. 2019), which further excludes many ditches. A critical step to improve future estimates of ditch GHG budgets is to map the global extent of ditches, particularly irrigation and urban canals. We are optimistic that recent developments in mapping using artificial intelligence (e.g., Habib et al. 2024; Lidberg et al. 2023) will help to close these knowledge gaps in the coming years. Given that ditches are anthropogenic features, constraining their GHG emissions is relevant for GHG national inventory reporting and achieving national goals for net‐zero carbon emissions.

### 4.2 Ditch Greenhouse Gas Emission Global Warming Potentials

Based on the widely used GWP_100_ metric, CO_2_ made the largest average contribution (51%), followed by CH_4_ (43.5%) and N_2_O (5.5%). While current IPCC methodology for reporting ditch emissions focuses on CH_4_, we find that CO_2_ and N_2_O to a lesser extent, also have important contributions when considering GWP. Where areal ditch CO_2_ emissions vary substantially from those of the adjacent land area, this suggests a need to develop methods and emission factors for ditch CO_2_ emissions. As a crude average from our collated dataset (without disaggregating data by climate zone or land use) a global “Tier 1” emission factor for ditch CO_2_ emissions would be 6.19 t CO_2_‐C ha^−1^ yr.^−1^ (95% CI: 4.61–7.76 t CO_2_‐C ha^−1^ yr.^−1^). Most studies only measure daytime GHG emissions, thus this estimate could be further refined by accounting for 27% higher night‐time CO_2_ emissions (Gómez‐Gener et al. 2021), resulting in 7.86 t CO_2_‐C ha^−1^ yr.^−1^. Despite its relatively lower contribution to total GHG emissions, N_2_O emissions from ditches may be notable at the landscape scale, particularly in agricultural catchments where they may disproportionately contribute to landscape‐scale N_2_O budgets (Outram and Hiscock 2012). When considering SGWP_100_/SGCP_100_, we find that CH_4_ dominates emissions (54%), although CO_2_ is not far behind (41%). Only two of the 22 studies we used for these estimates reported CH_4_ ebullition, therefore CH_4_ is likely underestimated. Especially considering that ebullition can make up a significant proportion (mean ± SD: 80% ± 17% from our dataset) of total CH_4_ emissions from ditches.

### 4.3 Complex Drivers of CO_2_ Emissions From Ditches

We did not observe an effect of land use type, climate zone, trophic state, soil type, hydrological regime, and vegetation presence on ditch CO_2_ emissions, likely because these variables do not fully capture the complex and interacting mechanisms affecting CO_2_ transport into ditches, in situ uptake, and release. The lack of a response of CO_2_ to broad climate zones and land use categories may be because local‐scale temporal, environmental, and/or management conditions may have greater impact. For example, within the agricultural land‐use class, management intensities vary from extensive grazing with little fertilization (Vermaat et al. 2011), to intensively farmed arable land with high fertilizer inputs (Harrison and Matson 2003). Additionally, variables that promote CO_2_ emission may also promote CO_2_ uptake, depending on the environmental conditions, resulting in high variability the net flux. For example, the type of vegetation present is important in determining the CO_2_ source/sink response. Lower CO_2_ emissions are often observed in algae‐dominated waterbodies compared to macrophyte‐dominated ones due to low oxygen concentrations lowering mineralization rates in the former (Brothers et al. 2013). Eutrophication can have divergent effects on gaseous carbon emissions, promoting net CO_2_ uptake through primary production (Vachon et al. 2020) or CO_2_ emission by stimulating respiration (Kortelainen et al. 2006). Although trophic status did not have a significant impact on ditch CO_2_ fluxes, emissions were moderately correlated with TN, and emissions from eutrophic ditches were highest on average. There were however also more occurrences of CO_2_ uptake in eutrophic ditches (3) than in oligotrophic ditches (0), leading to high overall variability. A similarly contrasting response in CO_2_ to nutrient status has been observed previously between large and small lakes (DelSontro et al. 2018). There, CO_2_ emissions were positively related to productivity in small systems, but negatively related to productivity in larger systems. This trend was attributed to nutrient and carbon inputs driving CO_2_ emission in small systems, while in larger systems nutrient inputs enhanced primary production, which tends to be more important in those systems (DelSontro et al. 2018).

There was no effect of intermittent drying on CO_2_ emissions from ditches, with similar emission rates from perennial, non‐perennial when water‐filled, and non‐perennial when dry. Drying can have a legacy effect of lower CO_2_ emissions once flow resumes in rivers (Silverthorn et al. 2024) and conversely cause an accumulation of CO_2_ in remnant persistent pools (Bretz et al. 2023) as well as CO_2_ emission pulses upon sediment rewetting (Arce et al. 2021; Looman et al. 2017). We did not observe evidence of these trends in ditches, likely because studies about the effects of drying cycles on ditch GHG emissions are still lacking. Ditches could also be more frequently exposed to drying than rivers, and microbial communities more frequently exposed to water stress can be more resilient and better adapted to changing hydrological conditions (Fierer et al. 2003). Dry inland water sediments can have notable CO_2_ emissions (Keller et al. 2020), making up 14 to 77% of total emissions in non‐perennial river networks (López‐Rojo et al. 2024). High emissions of CO_2_ from dry inland waters were attributed to oxygenation stimulating microbial activity and water no longer limiting the diffusion of gas, with areal emission rates higher than those from lentic waters (Keller et al. 2020). We observed similarly high CO_2_ emissions from dry ditches, with emission rates comparable to water‐filled ditches.

Increasingly, research is pointing to the importance of lateral GHG inputs in driving emissions (Rocher‐Ros et al. 2023), particularly in headwater streams—and presumably drainage ditches—where connectivity with the surrounding terrestrial ecosystem is high (Hotchkiss et al. 2015). Despite higher DOC concentrations in ditches draining organic soils than mineral soils, neither CO_2_ nor N_2_O emissions differed between catchment soil types. This may suggest that external GHG inputs are more influential than the internal metabolism of terrestrial carbon in ditches. Alternatively, the shallow waters and low flows in ditches promote high internal productivity (in contrast to streams), making up for the lower allochthonous organic carbon inputs to ditches draining mineral soils. Similar results have been observed in boreal forest ditches (Peacock, Granath, Wallin, Högbom, and Futter 2021). It is also possible that connectivity to organic soils is a more important driver of CH_4_ emissions than CO_2_ and N_2_O, due to anoxic conditions driving methanogenesis in wetlands (Rocher‐Ros et al. 2023).

### 4.4 Climate, Trophic State and Drying Influence Ditch N_2_O Emissions

Variations in N_2_O emissions from ditches were driven by climate and trophic state, and drying. The higher N_2_O emissions in temperate than continental climates may, in part, be explained by MAT, which had an apparently limiting effect on N_2_O in continental climates. However, in temperate climates, some other variables besides temperature must also be important, given the high variability in N_2_O emissions. In line with our expectations, N_2_O emissions were highest from eutrophic and hypereutrophic ditches, associated with greater nutrient inputs. This effect was corroborated by the positive correlation between N_2_O emissions and both TN and NO_3_^−^. Therefore, management strategies associated with reducing nitrogen inputs to ditches can be used to reduce their global warming impact. Despite this relationship with trophic state, we did not find the expected differences between land use types. We expected agricultural and urban land uses to have the highest N_2_O emissions due to nutrient‐rich fertilizer and wastewater inflows. Although N_2_O emissions from agricultural land uses were the highest on average, they were also the most variable, and did not significantly differ from other land uses. The lack of a clear effect of land use on N_2_O emissions may be, as with CO_2_, due to a gradient of management intensities within land use categories.

Perennial ditches had higher N_2_O emissions than non‐perennial (both water‐filled and dry). Research on GHG emissions from dry ditches (especially for N_2_O) is limited. Pulses of N_2_O have been observed following the rewetting of fine, carbon‐ and nitrogen‐rich, dry sediments in urban ditches (Gallo et al. 2014). The low N_2_O emissions from non‐perennial dry and water‐filled ditches we observed are possibly due to the negative effects of desiccation on microbial communities, which persist after flow resumption, previously observed in riverine sediments (Schreckinger et al. 2022). To better constrain global estimates of N_2_O emissions from ditches, the next challenge—beyond determining the extent of ditches and canals—will be to determine the prevalence of non‐perennial ditches worldwide, as has already been done for streams and rivers (Messager et al. 2021). The geographic gaps in our dataset (e.g., South America, Africa, and Asia) correspond to large regions with arid climates, presenting an important area of uncertainty that needs to be constrained for global ditch N_2_O emission estimates.

The IPCC methodology provides Tier 1 emission factors for estimating “indirect” emissions of N_2_O from water bodies, based on default conversion factors for the proportion of anthropogenic nitrogen applied to land (e.g., as fertilizer and manure) that will be converted to N_2_O in downstream aquatic ecosystems (De Klein et al. 2006; Lovelock et al. 2019). These default emission factors have high uncertainty and effectively assume a linear relationship between NO_3_^−^ leaching from land to water and subsequent N_2_O production (Webb et al. 2021). Although we did find that ditch N_2_O emissions moderately correlated with TN and NO_3_^−^, this is not always the case. The default emission factors also do not distinguish between different types of receiving waterbodies, for example, constructed ditches versus natural streams. Moreover, they are not disaggregated by land use, trophic state, climate zone, soil type, or other potentially important explanatory factors (Outram and Hiscock 2012; Peacock, Audet, Bastviken, Cook, et al. 2021). Our results suggest that there may be scope to refine the current Tier 1 methodology for N_2_O, potentially providing greater disaggregation as a function of using climate and/or the characteristics of the receiving waterbody.

### 4.5 Conclusions

Our preliminary estimates show that CO_2_ and N_2_O emissions from ditches are quantitively significant but largely unaccounted components of global inland water GHG budgets. It appears that CO_2_ and CH_4_ dominate overall ditch emissions (based on both GWP_100_ and SGWP_100_/SGCP_100_) but the emission of all three GHGs needs to be considered, especially given that ditches can have elevated GHG emissions compared to surrounding terrestrial environments. Although ditch N_2_O emission contributions were lower, the observed relationship with nutrient status indicates that management strategies aimed at reducing nitrogen‐rich inputs to ditches is likely to be an effective climate mitigation measure. Such strategies can include regional scale legislation regarding the use of fertilizers and manure as well as local‐scale measures such as installing fences to prevent cattle entering water (Malerba et al. 2022), creating vegetated riparian zones, dredging to remove sediments, increasing aeration, managing water levels, or even adding chemical that bind nutrients (Paranaíba and Kosten 2024). More research is needed to disentangle the complex and interactive controls on CO_2_ emissions from ditches, with a focus on under what conditions ditches act as a source or sink of CO_2_. The substantial unexplained variation in CO_2_ and N_2_O fluxes in ditches calls for more targeted measurements for an improved understanding of their drivers across scales. More studies examining seasonal variations in GHG emissions from ditches are needed to better constrain annual emission estimates. Given that ditches have higher areal CO_2_ and N_2_O emissions than many other inland waters, an improved estimate of the global extent of ditches will be the key to elucidating their global climate impact.

## Author Contributions

**Teresa Silverthorn:** data curation, formal analysis, funding acquisition, visualization, writing – original draft. **Joachim Audet:** data curation, writing – review and editing. **Chris D. Evans:** data curation, funding acquisition, writing – review and editing. **Judith van der Knaap:** data curation, writing – review and editing. **Sarian Kosten:** data curation, writing – review and editing. **Quinten Struik:** data curation, writing – review and editing. **Jackie Webb:** data curation, writing – review and editing. **Wenxin Wu:** data curation, writing – review and editing. **Zhifeng Yan:** data curation, writing – review and editing. **Mike Peacock:** conceptualization, data curation, funding acquisition, supervision, writing – review and editing. **José Paranaíba:** data compilation and revision of the manuscript draft.

## Conflicts of Interest

The authors declare no conflicts of interest.

## Supporting information


Data S1.

## Data Availability

The data that support the findings of this study and the programming code to replicate analyses are openly available in Zenodo at https://doi.org/10.5281/zenodo.14753050.
