# Supplementary material for: The Importance of Ditches and Canals in Global Inland Water CO2 and N2O Budgets
Source: Glob Chang Biol. 2025 Mar 7;31(3):e70079. doi: 10.1111/gcb.70079 (PMC11886762; doi:10.1111/gcb.70079)
Supplement: Supplementary file 1 — Data S1. [file GCB-31-e70079-s001.docx]

# Supporting Information for

**The importance of ditches and canals in global inland water CO_2_ and N_2_O budgets**

Teresa Silverthorn^1*^, Joachim Audet^2^, Chris D. Evans^3^, Judith van der Knaap^4^, Sarian Kosten^4^, José Paranaíba^4^, Quinten Struik^4^, Jackie Webb^5,6^, Wenxin Wu^7^, Zhifeng Yan^7^, Mike Peacock^1,8^

**Affiliations**

1. Department of Geography and Planning, School of Environmental Sciences, University of Liverpool, Liverpool, UK

2. Department of Ecoscience, Aarhus University, Aarhus, Denmark

3. UK Centre for Ecology & Hydrology, Bangor, United Kingdom

4. Department of Ecology, Radboud Institute for Biological and Environmental Sciences, Radboud University, Nijmegen, the Netherlands

5. School of Agriculture and Environmental Science, University of Southern Queensland, Toowoomba, Australia

6. Centre for Sustainable Agricultural Systems, University of Southern Queensland, Toowoomba, Australia

7. Institute of Surface Earth System Science, School of Earth System Science, Tianjin University, Tianjin, China

8. Department of Aquatic Sciences and Assessment, Swedish University of Agricultural Sciences, Uppsala, Sweden

* Corresponding author: Teresa Silverthorn teresa.silverthorn@gmail.com

**Supplementary Text 1**

We used the coordinates reported in the study, or if not provided, we estimated the coordinates from a provided map of the study site(s) or the given name of the study site(s). These coordinates were used to determine the Köppen Geiger climate zone of the study area using the *LookupCZ* function in the *kgc* R-package (Bryant et al., 2017).

We collected mean annual temperature (MAT) and precipitation (MAP) data for each study as a historic average unless only data for the study period was reported. If this climate data was not reported in the study, we used values from another study in a nearby location, or from the closest meteorological station with open data available from the European Centre for Medium-Range Weather Forecasts through climate-data.org.

If elevation (masl) of the study site(s) was not reported in the study, it was estimated in Google Earth using the reported GPS coordinates of the study ditch(es) (or as near as possible based on the available information).

If ditch width was not reported, we used a mean of several (at least 3) measurements of the study ditch width from Google Earth. In some cases, the provided coordinates were not sufficient to identify the specific study ditch(es), or the satellite imagery was difficult to interpret (e.g. trees covering the ditches), in which cases it was not possible to include an estimate of ditch width.

If the water velocity was described qualitatively rather than quantitatively, we interpolated these for numerical values. “Slow flow” (*n* = 2) was replaced with the 1% percentile of all the velocity values (0.013 m s^-1^). “Low flow” (*n* = 2) was replaced with the lowest recorded velocity (0.01 m s^-1^). “Low/no flow” (*n* = 3) and “sluggish flow” (*n* = 2) were replaced with a value half of the lowest reported velocity (0.005 m s^-1^). “Standing water” was replaced with 0 m s^-1^.

We used a simple classification of mineral (*n* = 58) or organic soil (*n* = 61) dominating the ditch catchment. We classified a soil as organic if it was peat soil or any soil with organic matter as the major portion of the surface soil horizons.

To determine the trophic state of a site, we used Table 1 from Smith et al. (1999), which was used as the basis for Table 7.11 of the 2019 IPCC Refinement (Lovelock et al., 2019) for determining trophic state. We used available total nitrogen (TN), total phosphorus (TP), chlorophyll-*a* (chl-*a*), and/or Secchi disk depth data to classify trophic states accordingly. In the rare occasions that variables from a single study pointed to different trophic states, we went with the “higher” trophic state (e.g. hypereutrophic over eutrophic). When quantitative data about the nutrient status was not available, we determined the trophic state based on site descriptions (e.g. sites with current or historic use of fertilizer were likely eutrophic and ombrotrophic bogs are considered nutrient-poor as they are rain-fed) or satellite imagery (i.e. sites in intensive agricultural or urban areas were likely to be eutrophic). We categorized our data into one of four trophic state categories: hypereutrophic (*n* = 34), eutrophic (*n* = 42), mesotrophic (*n* = 14), and oligotrophic (*n* = 14). We assigned NA (*n* = 15) when it was not possible to determine trophic state.

We determined the hydrological status of the study ditch based on site descriptions of water flow or measurements of water table depth. If a site was dry with no surface water flow for at least one day per year, it was classified as non-perennial (*n* = 51). If a site had surface water all year round, it was classified as perennial (*n* = 63). If we were unable to determine hydrological status, we assigned NA (*n* = 5).

We determined the presence (*n* = 41) or absence (*n* = 15) of in-stream vegetation (e.g. algae, macrophytes, sphagnum) from site descriptions and/or photos, when available (NA: *n* = 63).

**Supplementary Table 1.** Full description of the search terms employed in the Web of Science database.

| (drainage ditch* OR drainage canal* OR canal* OR ditch* OR irrigation canal* OR agricultural ditch*) AND (Carbon dioxide OR CO2 OR nitrous oxide OR N2O OR greenhouse gas*) |
| --- |

**Supplementary Table 2.** Full list of the information collected from each study.

| **Basic information**  Authors  Title  Publication Year  Journal  **Geographic information**  Country  Latitude  Longitude  Site name  Elevation (masl)  **Climatic information**  Mean annual temperature (°C) “MAT”  Mean annual precipitation (mm) “MAP”  Köppen Geiger climate zone (arid, continental, temperate, tropical)  **General study characteristics**  Mean channel width (m)  Mean water depth (m)  Mean velocity (m s^-1^)  Mean discharge (m^3^ s^-1^)  Catchment land use (agriculture, natural/forest, urban, wetland)  Catchment soil type (organic, mineral)  Trophic state (hypertrophic, eutrophic, mesotrophic, oligotrophic)  Hydrological regime (perennial, non-perennial)  In-stream vegetation presence/absence  **GHG fluxes**  Annual carbon dioxide flux (g CO_2_ m^-2^ yr^-1^)  Annual nitrous oxide flux (g N_2_O m^-2^ yr^-1^)  Annual diffusive methane flux (if CO_2_ and/or N_2_O is also measured) (g CH_4_ m^-2^ yr^-1^)  Annual ebullitive methane flux (if CO_2_ and/or N_2_O is also measured) (g CH_4_ m^-2^ yr^-1^)  Flux conversion notes (if flux rate was converted, how)  Method (chamber, concentration)  Sampling period  Sampling frequency  **Other variables**  Dissolved oxygen (mg L ^-1^)  pH  Electrical conductivity (µs cm^-1^)  Total phosphorus (mg L^-1^)  Total nitrogen (mg L^-1^)  Chlorophyll-*a* (mg L^-1^)  Nitrate (mg L^-1^)  Secchi depth (m) | | | | |  |
| --- | --- | --- | --- | --- | --- |
| **Supplementary Table 3.** Warming impacts of greenhouse gas emissions from ditches in carbon dioxide equivalents (CO_2_-eq) based on global warming potential (GWP) and sustained global warming potential (SGWP) | | | | | |
|  | GWP_100_^1^ | | g CO_2_-eq m^-2^ yr^-1^ (±SD) | % | |
| CO_2_ | - | | 2085 ± 3202 | 51 | |
| CH_4_ | 27 | | 1683 ± 3871 | 43.5 | |
| N_2_O | 273 | | 210 ± 530 | 5.5 | |
|  | SGWP_100_^2^ | SGCP_100_^2^ | g CO_2_-eq m^-2^ yr^-1^ (±SD) | % | |
| CO_2_ | - | - | 2085 ± 3202 | 41 | |
| CH_4_ | 45 | 203 | 1589 ± 3779 | 54 | |
| N_2_O | 270 | 349 | 201 ± 516 | 5 | |
| ^1.^ Forster et al. (2021); ^2.^ Neubauer & Megonigal, (2015) | | | | | |

| **Supplementary Table 4.** Mean ± standard deviation and sample size (*n*) of carbon dioxide (CO_2_) and nitrous oxide (N_2_O) emissions from ditches by land use, vegetation presence/absence, soil type, climate, trophic status, hydrological status, and sampling method. | | | | |
| --- | --- | --- | --- | --- |
|  | g CO_2_ m^-2^ y^-1^ | (*n*) | g N_2_O m^-2^ y^-1^ | (*n*) |
|  |  |  |  |  |
| Agriculture | 1920 ± 2090 | (46) | 1.26 ± 2.17 | (37) |
| Natural/Forest | 2770 ± 3780 | (26) | 0.291 ± 0.409 | (7) |
| Urban | 1990 ± 2660 | (13) | 0.0407 ± 0.0351 | (2) |
| Wetland | 1250 ± 998 | (14) | 0.130 ± 0.177 | (10) |
|  |  |  |  |  |
| Vegetation | 1980 ± 3160 | (39) | 0.918 ± 2.33 | (19) |
| No vegetation | 1820 ± 1410 | (14) | 0.520 ± 0.864 | (6) |
|  |  |  |  |  |
| Mineral | 2360 ± 3360 | (46) | 0.866 ± 1.56 | (30) |
| Organic | 1780 ± 1740 | (53) | 0.922 ± 2.14 | (26) |
|  |  |  |  |  |
| Arid | 1070 ± 1900 | (5) | 0.694 ± 1.11 | (6) |
| Continental | 1358 ± 1300 | (39) | 0.0886 ± 0.154 | (20) |
| Temperate | 2510 ± 3360 | (47) | 1.58 ± 2.41 | (27) |
| Tropical | 3370 ± 1990 | (8) | 0.430 ± 0.526 | (3) |
|  |  |  |  |  |
| Hypereutrophic | 1690 ± 1480 | (28) | 0.984 ± 1.48 | (16) |
| Eutrophic | 2960 ± 3770 | (33) | 1.34 ± 2.51 | (22) |
| Mesotrophic | 1580 ± 1800 | (12) | 0.42 ± 0.915 | (11) |
| Oligotrophic | 1330 ± 1800 | (11) | 0.00549 ± 0.0541 | (5) |
|  |  |  |  |  |
| Perennial | 2320 ± 3140 | (54) | 1.54 ± 2.33 | (30) |
| Non-perennial (with water) | 1800 ± 1870 | (41) | 0.134 ± 0.208 | (22) |
| Non-perennial (dry) | 2090 ± 1930 | (12) | 0.0933 ± 0.119 | (3) |
|  |  |  |  |  |
| Chamber | 1790 ± 1620 | (70) | 0.471 ± 1.18 | (33) |
| Concentration | 2830 ± 4510 | (23) | 0.970 ± 1.61 | (18) |
|  |  |  |  |  |

| **Supplementary Table 5.** Global estimates of average area-specific carbon dioxide (CO_2_) and nitrous oxide (N_2_O) emissions from inland waters by ecosystem type. | | | | | |
| --- | --- | --- | --- | --- | --- |
|  | g CO_2_ m^-2^ y^-1^ | |  | g N_2_O m^-2^ y^-1^ | |
| Ditches | 2057 ± 2623 | This review | Ditches | 0.89 ± 1.8 | This review |
| Rivers/ streams | 10,648 | Raymond et al. (2013) | Rivers/ streams | 1.13 | Zheng et al. (2022) |
| Ponds | 561 | Holgerson & Raymond (2016) | Ponds | 0.08 | Zheng et al. (2022) |
| Lakes | 289 | Raymond et al. (2013) | Lakes | 0.20 | Zheng et al. (2022) |
| Reservoirs | 451 | Deemer et al. (2016) | Reservoirs | 0.44 | Zheng et al. (2022) |


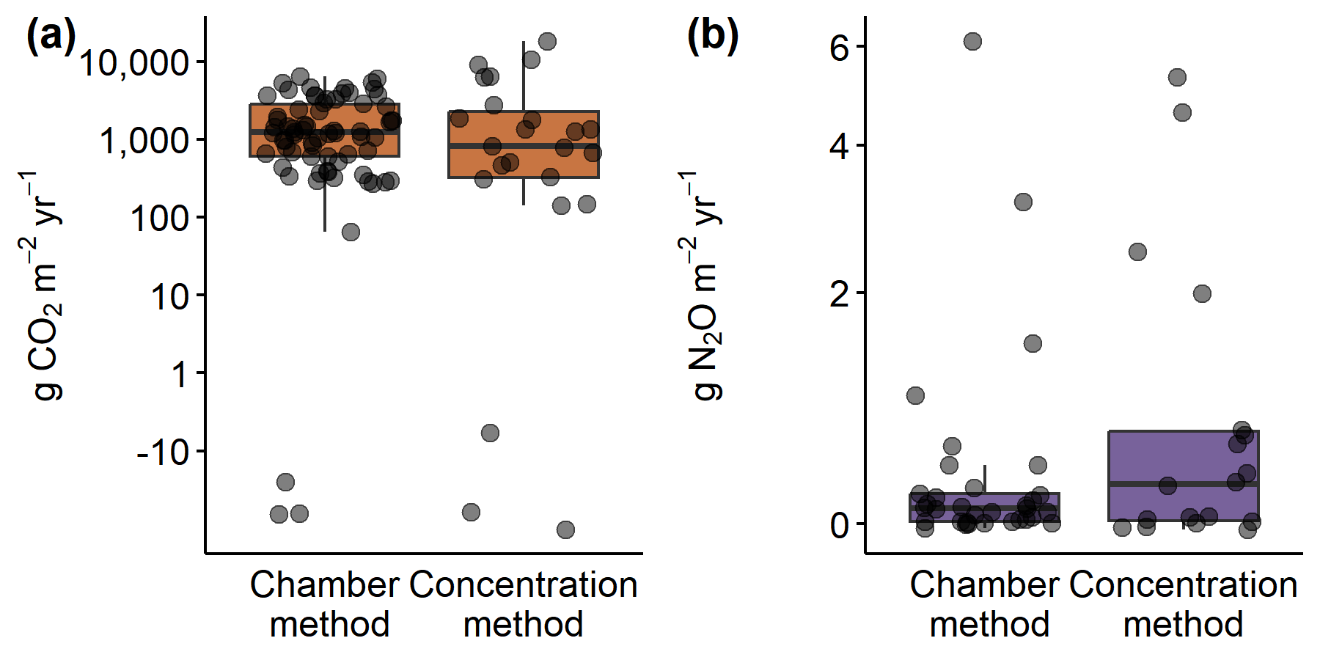


**Supplementary Figure 1.** Carbon dioxide and nitrous oxide emissions from global ditches did not differ between sampling methods (CO_2_: p = 0.58 and N_2_O: p = 0.43, Mann-Whitney U test).


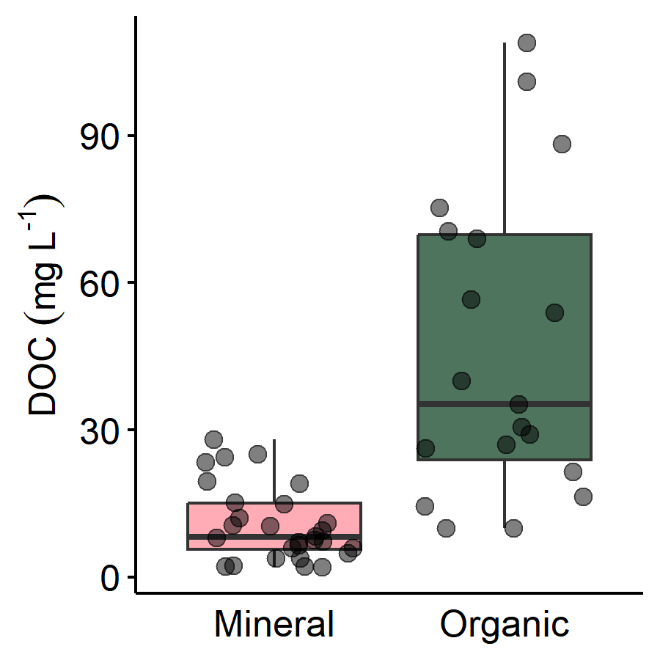


**Supplementary Figure 2.** Dissolved organic carbon (DOC) concentrations were higher in ditches draining organic than mineral soils (p < 0.0001, Mann-Whitney U test).


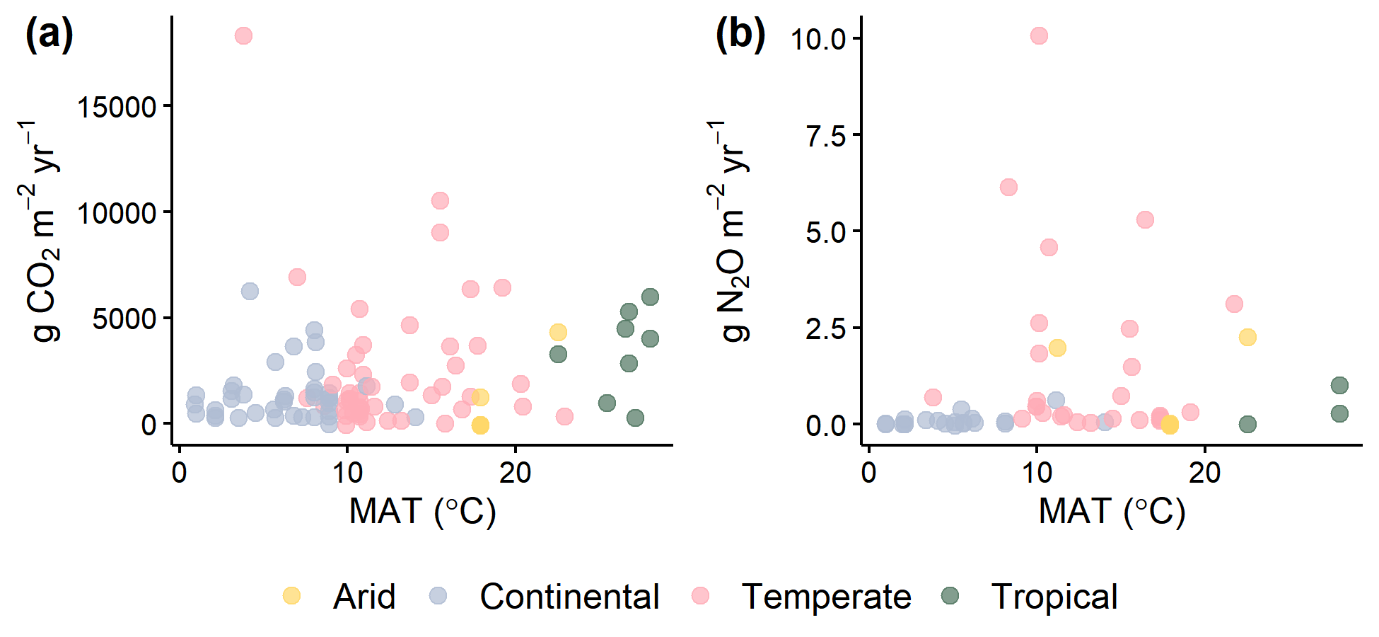


**Supplementary Figure 3.** Relationship between carbon dioxide (a) and nitrous oxide (b) emissions from global ditches and mean annual temperature (MAT; °C) (CO_2_: R = 0.15, p = 0.14; N_2_O: R = 0.25, p = 0.07).

**Supplementary References**

Bryant, C., Wheeler, N. R., Rubel, F., & French, R. H. (2017). *kgc: Koeppen-Geiger Climatic Zones* (Version 1.0.0.2) [Computer software]. https://cran.r-project.org/web/packages/kgc/index.html

Deemer, B. R., Harrison, J. A., Li, S., Beaulieu, J. J., DelSontro, T., Barros, N., Bezerra-Neto, J. F., Powers, S. M., dos Santos, M. A., & Vonk, J. A. (2016). Greenhouse Gas Emissions from Reservoir Water Surfaces: A New Global Synthesis. *BioScience*, *66*(11), 949–964. https://doi.org/10.1093/biosci/biw117

Forster, P., Storelvmo, T., Armour, K., Collins, W., Dufresne, J. L., Frame, D., Lunt, D. J., Mauritsen, T., Palmer, M. D., & Watanabe, M. (2021). Chapter 7: The earth’s energy budget, climate feedbacks, and climate sensitivity Climate Change 2021: The Physical Science Basis. In *Contribution of Working Group I to the Sixth Assessment Report of the Intergovernmental Panel on Climate Change* (p. 1017). Cambridge University Press. https://doi.org/10.1017/9781009157896.009

Holgerson, M. A., & Raymond, P. A. (2016). Large contribution to inland water CO_2_ and CH_4_ emissions from very small ponds. *Nature Geoscience*, *9*(3), 222–226. https://doi.org/10.1038/ngeo2654

Lovelock, C. E., Evans, C., Barros, N., Prairie, Y., Alm, J., Bastviken, D., Beaulieu, J. J., Garneau, M., Harby, A., Harrison, J., Pare, D., Raadal, H. L., Sherman, B., Zhang, C., Ogle, S. M., Grinham, A., Deemer, B., Aurelio dos Santos, M., Kosten, S., … Stepaneko, V. (2019). *Refinement to the 2006 IPCC Guidelines for National Greenhouse Gas Inventories, Volume 4: Agriculture, Forestry and Other Land Use (Chapter 7: Wetlands)*. IPCC.

Neubauer, S. C., & Megonigal, J. P. (2015). Moving Beyond Global Warming Potentials to Quantify the Climatic Role of Ecosystems. *Ecosystems*, *18*(6), 1000–1013. https://doi.org/10.1007/s10021-015-9879-4

Raymond, P. A., Hartmann, J., Lauerwald, R., Sobek, S., McDonald, C., Hoover, M., Butman, D., Striegl, R., Mayorga, E., Humborg, C., Kortelainen, P., Dürr, H., Meybeck, M., Ciais, P., & Guth, P. (2013). Global carbon dioxide emissions from inland waters. *Nature*, *503*(7476), 355–359. https://doi.org/10.1038/nature12760

Smith, V. H., Tilman, G. D., & Nekola, J. C. (1999). Eutrophication: Impacts of excess nutrient inputs on freshwater, marine, and terrestrial ecosystems. *Environmental Pollution*, *100*(1), 179–196. https://doi.org/10.1016/S0269-7491(99)00091-3

Zheng, Y., Wu, S., Xiao, S., Yu, K., Fang, X., Xia, L., Wang, J., Liu, S., Freeman, C., & Zou, J. (2022). Global methane and nitrous oxide emissions from inland waters and estuaries. *Global Change Biology*, *28*(15), 4713–4725. https://doi.org/10.1111/gcb.16233

**Data Sources**

Abulaiti, A., She, D., Pan, Y., Shi, Z., Hu, L., Huang, X., Shan, J., & Xia, Y. (2024). Drainage ditches are significant sources of indirect N_2_O emissions regulated by available carbon to nitrogen substrates in salt-affected farmlands. *Water Research*, *251*, 121164. https://doi.org/10.1016/j.watres.2024.121164

Ball, T., Smith, K. A., & Moncrieff, J. B. (2007). Effect of stand age on greenhouse gas fluxes from a Sitka spruce [ *Picea sitchensis* (Bong.) Carr.] chronosequence on a peaty gley soil. *Global Change Biology*, *13*(10), 2128–2142. https://doi.org/10.1111/j.1365-2486.2007.01427.x

Clark, L., Strachan, I. B., Strack, M., Roulet, N. T., Knorr, K.-H., & Teickner, H. (2023). Duration of extraction determines CO_2_ and CH_4_ emissions from an actively extracted peatland in eastern Quebec, Canada. *Biogeosciences*, *20*(3), 737–751. https://doi.org/10.5194/bg-20-737-2023

Daun, C., Huth, V., Gaudig, G., Günther, A., Krebs, M., & Jurasinski, G. (2023). Full-cycle greenhouse gas balance of a Sphagnum paludiculture site on former bog grassland in Germany. *Science of The Total Environment*, *877*, 162943. https://doi.org/10.1016/j.scitotenv.2023.162943

Davis, K., Santos, I. R., Perkins, A. K., Webb, J. R., & Gleeson, J. (2020). Altered groundwater discharge and associated carbon fluxes in a wetland-drained coastal canal. *Estuarine, Coastal and Shelf Science*, *235*, 106567. https://doi.org/10.1016/j.ecss.2019.106567

Deng, O., Li, X., Deng, L., Zhang, S., Gao, X., Lan, T., Zhou, W., Tian, D., Xiao, Y., Yang, J., Ou, D., & Luo, L. (2020). Emission of CO_2_ and CH_4_ from a multi-ditches system in rice cultivation region: Flux, temporal-spatial variation and effect factors. *Journal of Environmental Management*, *270*, 110918. https://doi.org/10.1016/j.jenvman.2020.110918

Deng, O., Ran, J., Gao, X., Lin, X., Lan, T., Luo, L., Xiong, Y., Liu, J., Ou, D., Fei, J., & Huang, R. (2023). CH_4_ and CO_2_ emissions in water networks of rice cultivation regions. *Environmental Research*, *218*, 115041. https://doi.org/10.1016/j.envres.2022.115041

Dhandapani, S., Girkin, N. T., & Evers, S. (2022). Spatial variability of surface peat properties and carbon emissions in a tropical peatland oil palm monoculture during a dry season. *Soil Use and Management*, *38*(1), 381–395. https://doi.org/10.1111/sum.12741

Fang, X., Wang, C., Xiao, S., Yu, K., Zhao, J., Liu, S., & Zou, J. (2023). Lower methane and nitrous oxide emissions from rice-aquaculture co-culture systems than from rice paddies in southeast China,. *Agricultural and Forest Meteorology*, *338*, 109540. https://doi.org/10.1016/j.agrformet.2023.109540

Green, S. M., Baird, A. J., Evans, C. D., Peacock, M., Holden, J., Chapman, P. J., & Smart, R. P. (2018). Methane and carbon dioxide fluxes from open and blocked ditches in a blanket bog. *Plant and Soil*, *424*(1), 619–638. https://doi.org/10.1007/s11104-017-3543-z

Gremmen, T., van Dijk, G., Postma, J., Colina, M., de Senerpont Domis, L. N., Velthuis, M., van de Haterd, R., Kuipers, F., van Rossum, H., Smolders, A. J. P., & Kosten, S. (2023). Factors influencing submerged macrophyte presence in fresh and brackish eutrophic waters and their impact on carbon emissions. *Aquatic Botany*, *187*, 103645. https://doi.org/10.1016/j.aquabot.2023.103645

Günther, A., Barthelmes, A., Huth, V., Joosten, H., Jurasinski, G., Koebsch, F., & Couwenberg, J. (2020). Prompt rewetting of drained peatlands reduces climate warming despite methane emissions. *Nature Communications*, *11*(1), 1644. https://doi.org/10.1038/s41467-020-15499-z

Harrison, J., & Matson, P. (2003). Patterns and controls of nitrous oxide emissions from waters draining a subtropical agricultural valley. *Global Biogeochemical Cycles*, *17*(3), 2002GB001991. https://doi.org/10.1029/2002GB001991

Hendriks, L., Weideveld, S., Fritz, C., Stepina, T., Aben, R. C. H., Fung, N. E., & Kosten, S. (2024). Drainage ditches are year‐round greenhouse gas hotlines in temperate peat landscapes. *Freshwater Biology*, *69*(1), 143–156. https://doi.org/10.1111/fwb.14200

Hyvönen, N. P., Huttunen, J. T., Shurpali, N. J., Lind, S. E., Marushchak, M. E., Heitto, L., & Martikainen, P. J. (2013). The role of drainage ditches in greenhouse gas emissions and surface leaching losses from a cutaway peatland cultivated with a perennial bioenergy crop. *Boreal Environment Research*, *18*, 109–126.

Järveoja, J., Peichl, M., Maddison, M., Teemusk, A., & Mander, Ü. (2016). Full carbon and greenhouse gas balances of fertilized and nonfertilized reed canary grass cultivations on an abandoned peat extraction area in a dry year. *GCB Bioenergy*, *8*(5), 952–968. https://doi.org/10.1111/gcbb.12308

Jauhiainen, J., & Silvennoinen, H. (2012). Diffusion GHG fluxes at tropical peatland drainage canal water surfaces. *Suoseura*, *63*(3–4), 93–105.

Klaus, M., Geibrink, E., Jonsson, A., Bergström, A.-K., Bastviken, D., Laudon, H., Klaminder, J., & Karlsson, J. (2018). Greenhouse gas emissions from boreal inland waters unchanged after forest harvesting. *Biogeosciences*, *15*(18), 5575–5594. https://doi.org/10.5194/bg-15-5575-2018

Macklin, P. A., Maher, D. T., & Santos, I. R. (2014). Estuarine canal estate waters: Hotspots of CO_2_ outgassing driven by enhanced groundwater discharge? *Marine Chemistry*, *167*, 82–92. https://doi.org/10.1016/j.marchem.2014.08.002

Manning, F. C., Kho, L. K., Hill, T. C., Cornulier, T., & Teh, Y. A. (2019). Carbon Emissions From Oil Palm Plantations on Peat Soil. *Frontiers in Forests and Global Change*, *2*, 37. https://doi.org/10.3389/ffgc.2019.00037

Matthews, R. A., Chadwick, D. R., Retter, A. L., Blackwell, M. S. A., & Yamulki, S. (2010). Nitrous oxide emissions from small-scale farmland features of UK livestock farming systems. *Agriculture, Ecosystems & Environment*, *136*(3), 192–198. https://doi.org/10.1016/j.agee.2009.11.011

McPhillips, L. E., Groffman, P. M., Schneider, R. L., & Walter, M. T. (2016). Nutrient Cycling in Grassed Roadside Ditches and Lawns in a Suburban Watershed. *Journal of Environmental Quality*, *45*(6), 1901–1909. https://doi.org/10.2134/jeq2016.05.0178

Otani, S., Yamanaka, R., Kozuki, Y., Fujishima, K., & Hirata, M. (2021). Spatial and temporal distribution of carbon dioxide flux in Amagasaki Canal, Japan. *Coastal Engineering Journal*, *63*(3), 422–432. https://doi.org/10.1080/21664250.2021.1964231

Palmia, B., Leonardi, S., Viaroli, P., & Bartoli, M. (2021). Regulation of CO_2_ fluxes along gradients of water saturation in irrigation canal sediments. *Aquatic Sciences*, *83*(1), 18. https://doi.org/10.1007/s00027-020-00773-5

Panneer Selvam, B., Natchimuthu, S., Arunachalam, L., & Bastviken, D. (2014). Methane and carbon dioxide emissions from inland waters in India – implications for large scale greenhouse gas balances. *Global Change Biology*, *20*(11), 3397–3407. https://doi.org/10.1111/gcb.12575

Peacock, M., Audet, J., Bastviken, D., Cook, S., Evans, C. D., Grinham, A., Holgerson, M. A., Högbom, L., Pickard, A. E., Zieliński, P., & Futter, M. N. (2021). Small artificial waterbodies are widespread and persistent emitters of methane and carbon dioxide. *Global Change Biology*, *27*(20), 5109–5123. https://doi.org/10.1111/gcb.15762

Peacock, M., Gauci, V., Baird, A. J., Burden, A., Chapman, P. J., Cumming, A., Evans, J. G., Grayson, R. P., Holden, J., Kaduk, J., Morrison, R., Page, S., Pan, G., Ridley, L. M., Williamson, J., Worrall, F., & Evans, C. D. (2019). The full carbon balance of a rewetted cropland fen and a conservation-managed fen. *Agriculture, Ecosystems & Environment*, *269*, 1–12. https://doi.org/10.1016/j.agee.2018.09.020

Pelsma, K. A. J., Verhagen, D. A. M., Dean, J. F., Jetten, M. S. M., & Welte, C. U. (2023). Methanotrophic potential of Dutch canal wall biofilms is driven by Methylomonadaceae. *FEMS Microbiology Ecology*, *99*(10), fiad110. https://doi.org/10.1093/femsec/fiad110

Pönisch, D. L., Breznikar, A., Gutekunst, C. N., Jurasinski, G., Voss, M., & Rehder, G. (2023). Nutrient release and flux dynamics of CO_2_, CH_4_, and N_2_O in a coastal peatland driven by actively induced rewetting with brackish water from the Baltic Sea. *Biogeosciences*, *20*(2), 295–323. https://doi.org/10.5194/bg-20-295-2023

Reay, D. S., Smith, K. A., & Edwards, A. C. (2003). Nitrous oxide emission from agricultural drainage waters. *Global Change Biology*, *9*(2), 195–203. https://doi.org/10.1046/j.1365-2486.2003.00584.x

Schrier-Uijl, A. P., Kroon, P. S., Hensen, A., Leffelaar, P. A., Berendse, F., & Veenendaal, E. M. (2010). Comparison of chamber and eddy covariance-based CO_2_ and CH_4_ emission estimates in a heterogeneous grass ecosystem on peat. *Agricultural and Forest Meteorology*, *150*(6), 825–831. https://doi.org/10.1016/j.agrformet.2009.11.007

Schrier-Uijl, A. P., Veraart, A. J., Leffelaar, P. A., Berendse, F., & Veenendaal, E. M. (2011). Release of CO_2_ and CH_4_ from lakes and drainage ditches in temperate wetlands. *Biogeochemistry*, *102*(1–3), 265–279. https://doi.org/10.1007/s10533-010-9440-7

Smukler, S. M., O’Geen, A. T., & Jackson, L. E. (2012). Assessment of best management practices for nutrient cycling: A case study on an organic farm in a Mediterranean-type climate. *Journal of Soil and Water Conservation*, *67*(1), 16–31. https://doi.org/10.2489/jswc.67.1.16

Smukler, S. M., Sánchez-Moreno, S., Fonte, S. J., Ferris, H., Klonsky, K., O’Geen, A. T., Scow, K. M., Steenwerth, K. L., & Jackson, L. E. (2010). Biodiversity and multiple ecosystem functions in an organic farmscape. *Agriculture, Ecosystems & Environment*, *139*(1), 80–97. https://doi.org/10.1016/j.agee.2010.07.004

Strack, M., & Zuback, Y. C. A. (2013). Annual carbon balance of a peatland 10 yr following restoration. *BIOGEOSCIENCES*, *10*(5), 2885–2896. https://doi.org/10.5194/bg-10-2885-2013

Sundh, I., Nilsson, M., Mikkelä, C., Granberg, G., & Svensson, B. H. (2000). Fluxes of Methane and Carbon Dioxide on Peat-mining Areas in Sweden. *AMBIO: A Journal of the Human Environment*, *29*(8), 499–503. https://doi.org/10.1579/0044-7447-29.8.499

Teh, Y. A., Silver, W. L., Sonnentag, O., Detto, M., Kelly, M., & Baldocchi, D. D. (2011). Large Greenhouse Gas Emissions from a Temperate Peatland Pasture. *Ecosystems*, *14*(2), 311–325. https://doi.org/10.1007/s10021-011-9411-4

Tian, L., Akiyama, H., Zhu, B., & Shen, X. (2018). Indirect NO_2_ emissions with seasonal variations from an agricultural drainage ditch mainly receiving interflow water. *Environmental Pollution*, *242*, 480–491. https://doi.org/10.1016/j.envpol.2018.07.018

Tian, L., Zhu, B., & Akiyama, H. (2017). Seasonal variations in indirect N_2_O emissions from an agricultural headwater ditch. *Biology and Fertility of Soils*, *53*(6), 651–662. https://doi.org/10.1007/s00374-017-1207-z

Tong, C. H. M., Nilsson, M. B., Sikström, U., Ring, E., Drott, A., Eklöf, K., Futter, M. N., Peacock, M., Segersten, J., & Peichl, M. (2022). Initial effects of post-harvest ditch cleaning on greenhouse gas fluxes in a hemiboreal peatland forest. *Geoderma*, *426*, 116055. https://doi.org/10.1016/j.geoderma.2022.116055

Vanags-Duka, M., Bārdule, A., Butlers, A., Upenieks, E. M., Lazdiņš, A., Purviņa, D., & Līcīte, I. (2022). GHG Emissions from Drainage Ditches in Peat Extraction Sites and Peatland Forests in Hemiboreal Latvia. *Land*, *11*(12), Article 12. https://doi.org/10.3390/land11122233

Vermaat, J. E., Hellmann, F., Dias, A. T. C., Hoorens, B., van Logtestijn, R. S. P., & Aerts, R. (2011). Greenhouse Gas Fluxes from Dutch Peatland Water Bodies: Importance of the Surrounding Landscape. *Wetlands*, *31*(3), 493–498. https://doi.org/10.1007/s13157-011-0170-y

Von Arnold, K., Weslien, P., Nilsson, M., Svensson, B. H., & Klemedtsson, L. (2005). Fluxes of CO_2_, CH_4_ and N_2_O from drained coniferous forests on organic soils. *Forest Ecology and Management*, *210*(1–3), 239–254. https://doi.org/10.1016/j.foreco.2005.02.031

Waddington, J. M., & Day, S. M. (2007). Methane emissions from a peatland following restoration. *Journal of Geophysical Research: Biogeosciences*, *112*(G3). https://doi.org/10.1029/2007JG000400

Wang, S., Wang, W., Liu, L., Zhuang, L., Zhao, S., Su, Y., Li, Y., Wang, M., Wang, C., Xu, L., & Zhu, G. (2018). Microbial Nitrogen Cycle Hotspots in the Plant-Bed/Ditch System of a Constructed Wetland with N_2_O Mitigation. *Environmental Science & Technology*, *52*(11), 6226–6236. https://doi.org/10.1021/acs.est.7b04925

Webb, J. R., Santos, I. R., Maher, D. T., Macdonald, B., Robson, B., Isaac, P., & McHugh, I. (2018). Terrestrial versus aquatic carbon fluxes in a subtropical agricultural floodplain over an annual cycle. *Agricultural and Forest Meteorology*, *260–261*, 262–272. https://doi.org/10.1016/j.agrformet.2018.06.015

Wu, W., Niu, X., Yan, Z., Li, S., Comer-Warner, S. A., Tian, H., Li, S.-L., Zou, J., Yu, G., & Liu, C.-Q. (2023). Agricultural ditches are hotspots of greenhouse gas emissions controlled by nutrient input. *Water Research*, *242*, 120271. https://doi.org/10.1016/j.watres.2023.120271

Xiao, Q., Hu, Z., Fu, C., Bian, H., Lee, X., Chen, S., & Shang, D. (2019). Surface nitrous oxide concentrations and fluxes from water bodies of the agricultural watershed in Eastern China. *Environmental Pollution*, *251*, 185–192. https://doi.org/10.1016/j.envpol.2019.04.076

Xiao, Q., Hu, Z., Hu, C., Islam, A. T., Bian, H., Chen, S., Liu, C., & Lee, X. (2021). A highly agricultural river network in Jurong Reservoir watershed as significant CO_2_ and CH_4_ sources. *Science of the Total Environment*, *769*, 144558.

Zhang, Y., Wang, X., Gong, X., Wu, S., Yuan, X., Liu, T., & Hou, C. (2021). Greenhouse gases concentrations and emissions in different inland water bodies in Chengdu Plain. *Desalination and Water Treatment*, *239*, 101–117. https://doi.org/10.5004/dwt.2021.27800
